# TRPV2 in muscle satellite cells is crucial for skeletal muscle remodelling

**DOI:** 10.1038/s41419-025-08242-3

**Published:** 2025-12-15

**Authors:** Yanzhu Chen, Kimiaki Katanosaka, Makoto Shibuya, Yubing Dong, Lidan Zhang, Motoi Kanagawa, So-ichiro Fukada, Keiji Naruse, Yuki Katanosaka

**Affiliations:** 1 Department of Cardiovascular Physiology, Graduate School of Medicine, Dentistry and Pharmaceutical Sciences, Okayama University, Okayama, Japan; 2 Department of Pharmacy, Kinjo Gakuin University, Nagoya, Aichi Japan; 3 Department of Biomedical Sciences, College of Life and Health Sciences, Chubu University, Kasugai, Aichi Japan; 4 Laboratory of Stem Cell Regeneration and Adaptation, Graduate School of Pharmaceutical Sciences, The University of Osaka, Suita, Osaka Japan; 5 Center for Medical Epigenetics, School of Basic Medical Sciences, Chongqing Medical University, Chongqing, China; 6 Department of Cell Biology and Molecular Medicine, Ehime University Graduate School of Medicine, Toon, Ehime Japan

**Keywords:** Cellular imaging, Experimental models of disease

## Abstract

Skeletal muscle remodelling relies on muscle stem cells (MuSCs) for regeneration after injury and hypertrophy in response to mechanical loading. However, the mechanisms that trigger MuSC activation and proliferation remain unclear. Transient receptor potential vanilloid 2 (TRPV2) ion channels respond to insulin-like growth factor-1 and mechanical stimuli to regulate the biological characteristics of various cells. Using a temporally inducible MuSC-specific conditional knockout (cKO) mouse, we show that TRPV2 regulates MuSC function and is essential for muscle remodelling. In cultured myofibre, MuSCs express TRPV2 and exhibit Ca^2+^ responses to the TRPV2 agonists 2-aminoethoxydiphenyl borate and probenecid, which are abolished upon TRPV2 deletion. TRPV2-deficient MuSCs exhibit reduced paired box 7 (Pax7) expression and impaired proliferation, suggesting TRPV2 is a factor that regulates the early stage of MuSC function. Myotube formation in MuSCs was enhanced by overexpression of TRPV2 and suppressed by TRPV2 deficiency, suggesting that TRPV2 is a factor that promotes myogenesis. Muscle-administered cardiotoxin promoted muscle regeneration and resulted in the appearance of numerous Pax7-positive MuSCs between myofibres. MuSC-specific *TRPV2* cKO mice exhibit substantially impaired muscle regeneration after cardiotoxin-induced injury, drastically reducing Pax7-positive MuSCs between myofibres. In floxed mice, mechanical loading via synergist ablation induces hypertrophy and greatly increases the number of myonuclei per myofibre. In contrast, MuSC-specific *TRPV2* cKO mice show no changes in myofibre thickness or nuclear number, either at baseline or after mechanical loading. Mechanical loading of floxed mice increased TRPV2^+^/Pax7^+^ double-positive MuSCs, but MuSC-specific *TRPV2* cKO mice showed no change. Additionally, MuSCs exhibit Ca^2+^ responses to hypo-osmotic stimuli, which are suppressed by TRPV2 inhibitors and TRPV2 deletion, suggesting that MuSCs exhibit TRPV2-dependent mechanical responses. These results establish TRPV2 as a critical regulator of MuSC-mediated muscle remodelling, an important finding that may lead to therapeutic strategies for muscle repair and adaptation.

## Introduction

Skeletal muscles maintain homoeostasis by adapting their form and function in response to external stress [1, 2]. This plasticity is supported by mature myofibres and their associated resident muscle stem cells (MuSCs) [3–6]. In adult skeletal muscle, MuSCs remain mitotically quiescent under resting conditions but become activated in response to muscle injury or exercise-induced mechanical loading. Upon activation, MuSCs provide essential myoblasts for muscle remodelling, including hypertrophy and regeneration [7, 8]. However, the mechanisms by which MuSCs detect and respond to muscle damage or stress remain unclear.

Ca^2+^ is a ubiquitous intracellular signal that regulates diverse cellular processes [9]. In skeletal muscle, Ca^2+^ signalling governs myogenesis, growth, regeneration, and the maintenance of contraction and plasticity [4]. Excitation–contraction coupling in muscle depends on intracellular Ca^2+^ dynamics mediated by interactions between ryanodine receptors and L-type voltage-gated Ca^2+^ channels, also known as dihydropyridine receptors [10]. The internal Ca^2+^ storage sensor stromal interaction molecule 1 (STIM1) and the store-operated calcium channel Orai1 regulate Ca^2+^ flux during muscle differentiation [11, 12]. Inositol trisphosphate (IP_3_) receptors mediate Ca^2+^ release during the early stages of muscle differentiation, whereas ryanodine receptors play a greater role as the sarcoplasmic reticulum matures [4]. Elucidation of Ca^2+^ signalling in MuSCs is needed to understand the processes that underlie muscle formation, growth, and regeneration.

Physical and chemical stimuli activate MuSCs and induce their proliferation. IGF-1 facilitates MuSC proliferation and promotes muscle growth via IP_3_ receptors and ryanodine receptors [4]. Additionally, physical stress triggers an extracellular Ca^2+^ response that enhances MuSC proliferation, suggesting the involvement of mechanosensor-mediated mechanotransduction pathways [13]. A recent study demonstrated delayed muscle regeneration in mice lacking the mechanosensitive channel Piezo1, indicating that Piezo1 regulates MuSC function [14].

In addition to Piezo1, members of the transient receptor potential (TRP) family of cation channels, which are candidate mechanosensors, are expressed in MuSCs [15]. However, whether TRP canonical family type 1 (TRPC1), which promotes MyoD expression through elevated intracellular Ca^2+^, exhibits mechanosensitivity remains controversial [16]. Previously, we reported that recombinant TRP vanilloid family type 2 (TRPV2) channels can be activated by hypotonicity- or stretch-induced mechanical loading in ectopic expression systems [17]. In skeletal muscle, TRPV2 is strongly localised at the sarcolemma of dystrophin-deficient *mdx* mice and δ-sarcoglycan-deficient BIO14.6 hamsters [18]. Although animal models expressing dominant-negative TRPV2 demonstrated amelioration of pathological features [19], the roles of TRPV2 in MuSC activation, proliferation, and fusion are unknown.

In this study, we generated MuSC-specific *TRPV2* conditional knockout (cKO) mice to investigate the physiological function of TRPV2 in MuSCs. TRPV2-deficient MuSCs have reduced expression of Pax7 and impaired proliferation, suggesting that TRPV2 regulates an early stage of MuSCs function. Myotube formation in MuSCs was dependent on the expression level of TRPV2, suggesting that TRPV2 contributes to the promotion of myogenesis. MuSC-specific *TRPV2* cKO mice also showed significantly attenuated muscle regeneration after cardiotoxin-induced injury and a reduced hypertrophic response to mechanical loading. Additionally, TRPV2 in MuSCs was essential for Ca^2+^ responses induced by mechanical loading. These findings indicate that TRPV2 in MuSCs is crucial for skeletal muscle remodelling.

## Results

### TRPV2 is expressed in mature muscle fibres and MuSCs

**Fig. 1:**
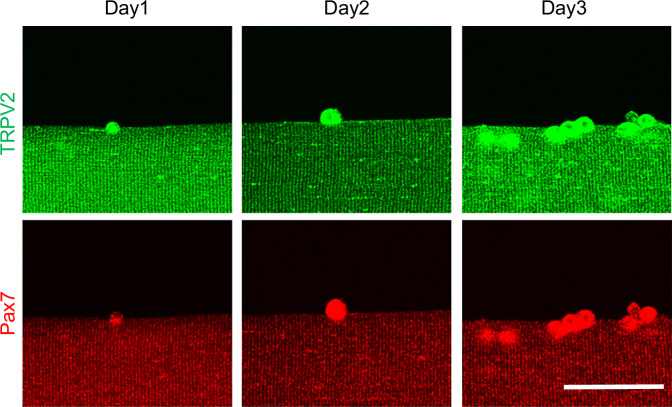
Expression of TRPV2 in cultured myofibres. Representative immunofluorescence staining of TRPV2 (green) and Pax7 (red) in cultured myofibres from floxed mice. Scale bar, 50 µm.

To examine TRPV2 expression in MuSCs, we isolated muscle fibres from the extensor digitorum longus (EDL) muscle of adult mice; these fibres were cultured for 3 days, then co-stained with an anti-TRPV2 antibody and an anti-Pax7 antibody (a specific marker of quiescent and activated satellite cells) [20] (Fig. 1). TRPV2 was strongly expressed in Pax7-positive cells attached to muscle fibres and cells in the T-tubules and sarcolemma of mature muscle fibres (Fig. 1). Thus, TRPV2 is expressed in MuSCs.

### Generation of MuSC-specific *TRPV2* cKO mice

**Fig. 2:**
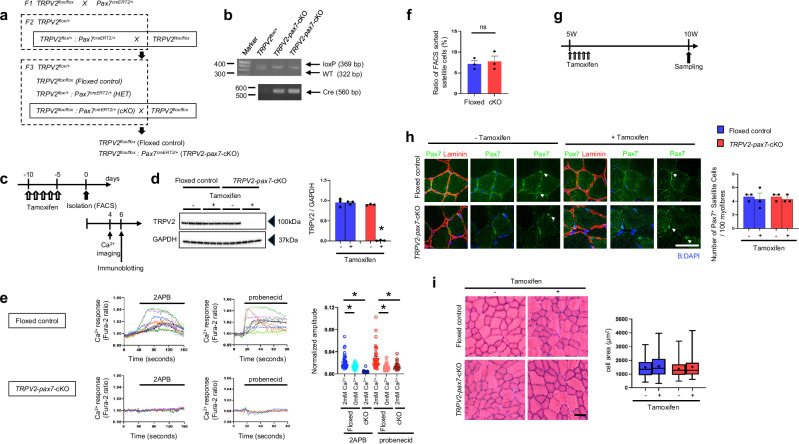
Generation of muscle satellite cell (MuSC)-specific TRPV2-deficient mice. **a** Crossbreeding strategy for the generation of TRPV2 conditional knockout (cKO) mice. Littermates from generation F3 were used as controls and cKO mice in all experiments. **b** Genotyping PCR of mouse genomic DNA. Upper panel: PCR fragments including the *loxP* insertion site within the *TRPV2* locus. The short fragment (322 bp) represents the wild-type *TRPV2* gene, whereas the longer fragment (369 bp) corresponds to the *loxP* insertion. Lower panel: PCR fragments of the *Cre* recombinase gene. **c** Timeline for TRPV2-deficiency induction, myofibre isolation, FACS sorting of MuSCs, Ca^2+^ imaging, and sampling. **d** Representative immunoblot of TRPV2 and GAPDH in cultured MuSCs from floxed or cKO mice, with or without tamoxifen treatment. **e** Representative traces of Ca^2+^ fluctuations in MuSCs isolated from floxed (upper panels) or cKO mice (lower panels) in response to 2-aminoethoxydiphenyl borate (2-APB) and probenecid in the presence of 2 mM extracellular Ca^2+^ (*n* = 18–59 cells from three mice per group). **f** The ratio of FACS-sorted satellite cells from tamoxifen-treated floxed and cKO myofibre. **g** Timeline of TRPV2-deficiency induction and tissue sampling. **h** Representative immunofluorescence images showing Pax7 (green), laminin (red), and 4′,6-diamidino-2-phenylindole (DAPI; blue) in MuSCs of the tibialis anterior (TA) muscle from 10-week-old mice. White arrowheads indicate Pax7^+^ MuSCs. **i** Representative haematoxylin and eosin staining and quantification of myofibre cross-sectional area in TA muscle from floxed and cKO mice (lower panels, *n* = 181–204 fibres from three mice per group). Centre line = median; + = mean; box limits = upper and lower quartiles; whiskers = minimum and maximum. Data are presented as mean ± s.e.m. *P < 0.05 (Tukey–Kramer test for **d**, **e**). Scale bar, 50 µm.

To examine the effect of TRPV2 deficiency in MuSCs, we crossed *TRPV2*^*flox/flox*^ (floxed-*TRPV2*) mice [21] with a transgenic line *Pax7*^*CreERT2*/*+*^ mouse line [22, 23] (Fig. 2a). Genomic DNA extracted from the tails of littermates was analysed by PCR to identify cKO mice carrying both the *loxP* insertion site within the *TRPV2* locus and the *Cre* recombinase gene (Fig. 2b). This approach generated MuSC-specific TRPV2-deficient mice in a tamoxifen-dependent manner (*TRPV2*-*Pax7*-cKO). To confirm the reduced expression of TRPV2 in MuSCs of *TRPV2*-*Pax7*-cKO mice, 12-week-old floxed and cKO mice were administered tamoxifen for 5 days. Five days after the final tamoxifen dose, muscle fibres were isolated from the EDL and cultured for 6 days to collect samples for immunoblotting (Fig. 2c). TRPV2 expression was clearly observed in the control group but was undetectable in tamoxifen-treated *TRPV2*-*Pax7*-cKO MuSCs (Fig. 2d, original data-1). MuSCs cultured for more than 4 days were suitable for Ca^2+^ imaging under fluid exchange conditions (Fig. 2c). To confirm the loss of TRPV2 function in TRPV2-deficient MuSCs, we analysed Ca^2+^ responses to 2-aminoethoxydiphenyl borate and probenecid, both activators of TRPV2 (Fig. 2e). MuSCs isolated from the EDL of tamoxifen-treated floxed-*TRPV2* mice exhibited an extracellular Ca^2+^-dependent Ca^2+^ response to these activators, whereas MuSCs from tamoxifen-treated *TRPV2*-*Pax7*-cKO mice showed no detectable Ca^2+^ response (Fig. 2e). These findings indicate that MuSCs derived from tamoxifen-treated *TRPV2*-*Pax7*-cKO mice are molecularly and functionally deficient in TRPV2.

The number of fleshly isolated MuSCs by fluorescence-activated cell sorting (FACS) does not differ between the tamoxifen-treated Floxed and cKO models (Fig. 2f). To assess the impact of TRPV2 deletion on MuSC maintenance, tamoxifen was administered to 5-week-old mice, and muscles were analysed at adulthood (10 weeks old) (Fig. 2g). The results showed no difference in the number of Pax7-positive cells between floxed-*TRPV2* mice and *TRPV2-Pax7*-cKO mice (Fig. 2h). No tissue damage or abnormalities were observed in TRPV2-deficient muscle, similar to the control group (Fig. 2i). These findings suggest that TRPV2 is not involved in the maintenance of MuSCs.

### Impairment of the early stages of myogenic progression in TRPV2-deficient MuSCs

**Fig. 3:**
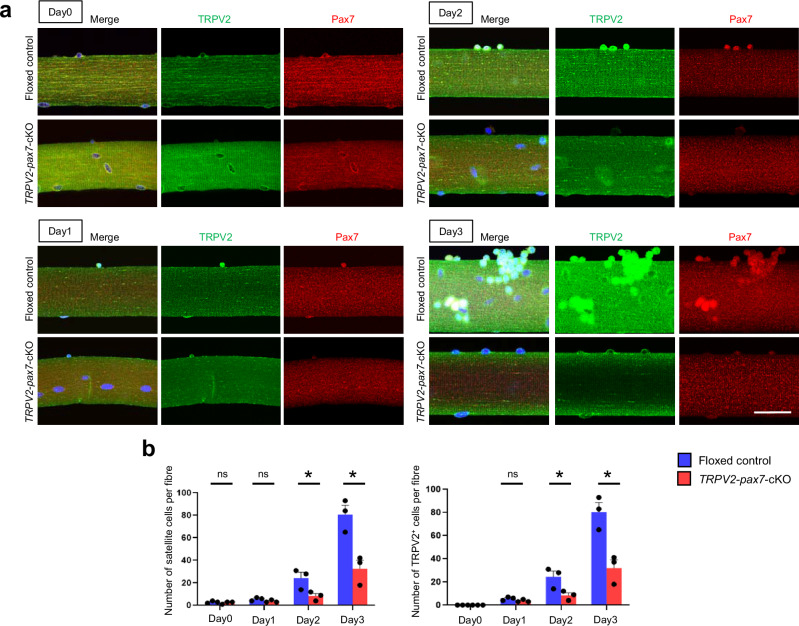
Decreased in Pax7-positive MuSC by TRPV2-deficiency. **a** Representative immunofluorescence staining of TRPV2 (green), Pax7 (red), and DAPI (blue) in MuSCs derived from myofibres isolated from the extensor digitorum longus (EDL) muscle of tamoxifen-treated floxed and cKO mice after 3 days in culture. **b** Quantification of satellite cell numbers (Pax7^+^ cells) and TRPV2^+^ cells in cultured floxed and TRPV2-deficient MuSCs. Data are presented as mean ± s.e.m. *P < 0.05 between the indicated groups (Student’s *t*-test). Scale bar, 50 µm.

To examine the effect of TRPV2 deficiency on myogenic activation, we cultured EDL fibres from tamoxifen-treated floxed and *TRPV2*-*Pax7*-cKO mice in growth medium for 3 days. Immunocytochemistry was performed to analyse the expression profiles of TRPV2 and Pax7 involved in the early stages of myogenic progression within MuSCs proliferating at the periphery of myofibres (Fig. 3). In tamoxifen-treated floxed-control fibres, the number of MuSCs (Pax7-positive cells) is increasing with each passing day (Fig. 3a, b). Since TRPV2 is expressed in Pax7-positive cells, the number of TRPV2-positive cells increased as well (Fig. 3a, b). In contrast, significantly fewer satellite cells were observed around myofibres isolated from TRPV2-deficient mice (Fig. 3a, b). Although the remaining TRPV2-positive cells were also Pax7 positive, their number is noticeably less than that of floxed-control fibres (Fig. 3a, b). These findings indicate that TRPV2 is a factor that regulates the early stage of MuSC function.

### Decrease in the proliferative capacity of TRPV2-deficient MuSCs

**Fig. 4:**
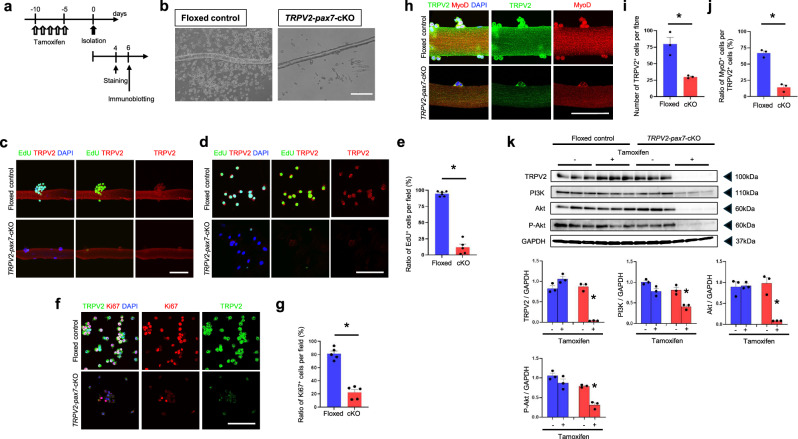
Impaired cell cycle entry in *TRPV2-Pax7*-cKO MuSCs. **a** Timeline for TRPV2-deficiency induction, cell isolation, and staining. **b** Representative images of proliferating satellite cells derived from myofibres isolated from the EDL muscle of floxed and TRPV2 cKO mice after 4 days in culture. **c** Representative EdU incorporation assay (green) in MuSCs located **c** on myofibres and **d** around myofibres. TRPV2 (red) and DAPI (blue) staining were performed simultaneously. **d** Representative immunofluorescence staining of EdU (green), TRPV2 (red), and DAPI (blue) in MuSCs derived from myofibres isolated from the EDL muscle of floxed and TRPV2 cKO mice after 4 days in culture. **e** Ratio of EdU^+^ cells. **f** Representative immunofluorescence staining of TRPV2 (green), Ki67 (red), and DAPI (blue) in MuSCs derived from myofibres isolated from the EDL muscle of floxed and TRPV2 cKO mice after 4 days in culture. **g** Ratio of Ki67^+^ cells. **h** Representative immunofluorescence staining of TRPV2 (green), MyoD (red), and DAPI (blue) in MuSCs derived from myofibres isolated from the EDL muscle of floxed and *TRPV2-Pax7*-cKO mice after 4 days in culture. **i** Quantification of TRPV2^+^ cells per fibre. **j** Ratio of MyoD^+^ cells among TRPV2^+^ cells. **k** Representative immunoblot showing PI3K, Akt, and phosphorylated Akt (P-Akt) expression in cultured MuSCs from floxed and cKO mice, with or without tamoxifen treatment. Data are presented as mean ± s.e.m. *P < 0.05 between the indicated groups (Student’s *t*-test for **e**, **g**, **i**, **j**). ^#^*P* < 0.05 (Tukey–Kramer test for **k**). Scale bar, 100 µm.

To examine the role of TRPV2 in MuSC proliferation, EDLs from tamoxifen-treated floxed-*TRPV2* and *TRPV2*-*Pax7*-cKO mice were cultured for 4 days (Fig. 4a, b). Numerous MuSCs were observed around EDLs isolated from floxed-control mice, whereas only a few were detected in *TRPV2*-*Pax7*-cKO mice (Fig. 4b). To assess the mitotic potential of TRPV2-deficient MuSCs, a 5-ethynyl-2′-deoxyuridine (EdU) incorporation assay was performed after 4 days of culture (Fig. 4c, d). Floxed myofibre-derived MuSCs exhibited EdU-positive cells both on and around the myofibres, which also showed TRPV2 expression (Fig. 4c, d, upper panels). In contrast, TRPV2-deficient MuSCs exhibited EdU-negative cells both on and around the myofibres (Fig. 4c, d, lower panels, Fig. 4e). Additionally, Ki67, a marker of proliferative potential [24], was expressed at significantly lower levels in TRPV2-deficient MuSCs (Fig. 4f, g), indicating impaired cell cycle entry. Double staining with anti-TRPV2 and anti-MyoD antibodies showed that most cells growing on floxed-control fibres were MuSCs (Fig. 4h–j). TRPV2 deficiency impaired the proliferation of MyoD-positive MuSCs. Furthermore, PI3K/AKT pathway activity was significantly attenuated in MuSCs cultured from myofibres that had been isolated from *TRPV2*-*Pax7*-cKO mice (Fig. 4k, original data-2). These findings suggest that TRPV2 regulates MuSC proliferation via the PI3K/AKT pathway. These findings demonstrate that TRPV2 is essential for MuSC proliferation.

### Impairment of myogenic fusion in TRPV2-deficient MuSCs

**Fig. 5:**
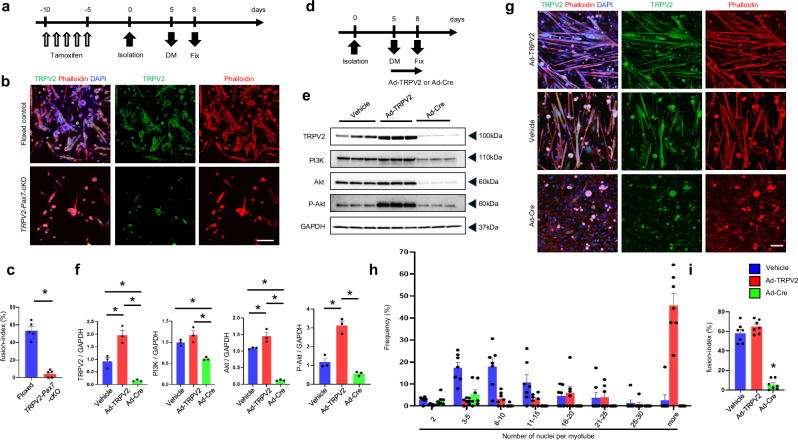
TRPV2 expression-dependent fusion of MuSCs. **a** Timeline for TRPV2-deficiency induction, myofibre isolation, medium replacement with differentiation medium, and fixation. **b** Representative immunofluorescence staining of TRPV2 (green), phalloidin (red), and DAPI (blue) in MuSCs derived from myofibres isolated from the EDL muscle of floxed and TRPV2 cKO mice after 4 days in culture. **c** Quantification of the fusion index. **d** Timeline for myofibre isolation, adenoviral infection, medium replacement, and fixation. **e**, **f** Representative immunoblots of TRPV2, PI3K, Akt, P-Akt, and GAPDH expression in MuSCs cultured in differentiation medium after infection with Ad-TRPV2 or Ad-Cre (*n* = 3 per group, *N* = 3 mice). **g** Representative immunofluorescence staining of TRPV2 (green), phalloidin (red), and DAPI (blue) in MuSCs infected with Ad-TRPV2 or Ad-Cre. **h** Quantification of the number of nuclei per single myotube in MuSCs transfected with Ad-TRPV2 (red) or Ad-Cre (green). **i** Quantification of the fusion index. Data are presented as mean ± s.e.m. *P < 0.05 (Student’s *t*-test for **c**; Tukey–Kramer test for **f**, **i**). Scale bar, 100 µm.

To examine the effect of TRPV2 deficiency on myogenic fusion, MuSCs derived from the EDLs of tamoxifen-treated *TRPV2*-*Pax7*-cKO mice were cultured in growth medium for 5 days, then transferred to differentiation medium for the induction of myogenic fusion (Fig. 5a). Compared with floxed-control MuSCs, myogenic fusion was significantly impaired in TRPV2-deficient MuSCs (Fig. 5b, c). Because the reduction in myogenic fusion was suspected to result from impaired MuSC proliferation in TRPV2-deficient MuSCs, cell numbers were maintained at equal levels until immediately before the switch to differentiation medium using cultured MuSCs from floxed-control myofibre (Fig. 5d–i). At this stage, TRPV2 expression in floxed-MuSCs was altered by adenoviral infection (Fig. 5d–f). Infection with Ad-TRPV2 increased TRPV2 expression approximately twofold, whereas Ad-Cre infection reduced expression to ~15% of the control level (Fig. 5e, f, original data-3). Ad-TRPV2-infected floxed-MuSCs exhibited a substantial increase in the number of nuclei per myotube, although the fusion index was unaffected (Fig. 5g, upper panels; Fig. 5h, i). In contrast, Ad-Cre-infected MuSCs showed substantial decreases in the number of nuclei per myotube and the fusion index (Fig. 5g, lower panels; Fig. 5h, i). These findings indicate that TRPV2 in MuSCs regulates myogenic fusion. In this experimental system, similar to the findings in Fig. 4k, PI3K/AKT pathway activity in MuSCs was dependent on TRPV2 expression (Fig. 5e), suggesting that TRPV2-mediated activation of the PI3K/AKT pathway is also critical for myogenic differentiation of MuSCs.

### Impairment of muscle regeneration after cardiotoxin-induced injury in MuSC-specific TRPV2-deficient mice

**Fig. 6:**
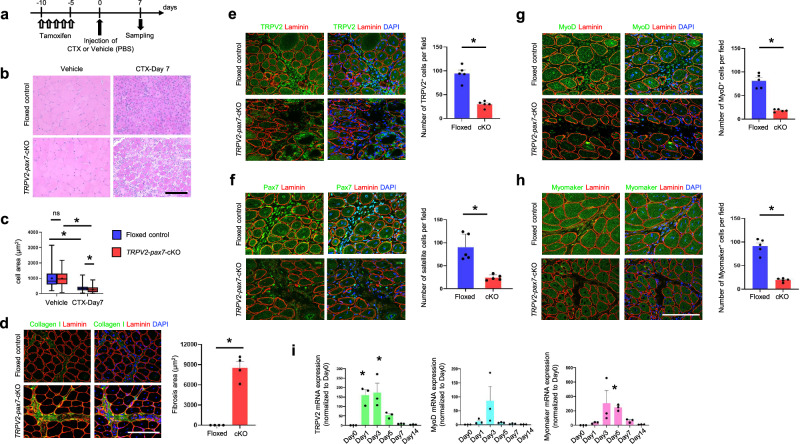
Impaired regeneration after cardiotoxin-induced muscle injury in MuSC-specific TRPV2-deficient mice. **a** Timeline for TRPV2-deficiency induction, cardiotoxin injection, and tissue sampling. **b** Representative haematoxylin and eosin staining of TA muscle. **c** Quantification of cross-sectional area from paraffin-embedded TA muscle sections (*n* = 412–463 cells from three mice per group). Centre line = median; + = mean; box limits = upper and lower quartiles; whiskers = minimum and maximum. **d** Representative immunofluorescence staining of collagen I (green), laminin (red), and DAPI (blue) in TA muscle from floxed and cKO mice. Quantification of collagen I-positive area (*n* = 4 sections from four TA muscles per group). **e–h** Representative immunofluorescence staining of TRPV2 (green), Pax7 (green), MyoD (green), or Myomaker (green), together with laminin (red) and DAPI (blue), in TA muscle from floxed and cKO mice. Quantification of TRPV2^+^, Pax7^+^, MyoD^+^, or Myomaker^+^ cells per field (*n* = 5 sections from three TA muscles per group). **i** mRNA expression levels of *TRPV2*, *MyoD*, and *Myomaker* on days 0, 1, 3, 5, 7, and 14 of culture (*n* = 3 mice per group). Data are presented as mean ± s.e.m. *P < 0.05 between the indicated groups (Student’s *t*-test for **f**, **h**; Tukey–Kramer test for **c**, **i**). Scale bar, 100 µm.

To elucidate the role of TRPV2 in MuSCs in vivo, we assessed muscle regeneration after cardiotoxin-induced injury in tamoxifen-treated *TRPV2*-*Pax7*-cKO mice (Fig. 6). In cardiotoxin-treated floxed-control mice, regenerating myofibres with central nuclei were observed after 7 days, but their diameters were significantly smaller than those in vehicle-treated controls (Fig. 6a–c). Conversely, individual myofibres were thinner in *TRPV2*-*Pax7*-cKO mice than in floxed-control mice on day 7 (Fig. 6b, c), and the collagen I-positive area was increased (Fig. 6d), suggesting a considerable delay in muscle regeneration.

To characterise satellite cells located between myofibres, we performed immunocytochemistry using anti-TRPV2, anti-Pax7, anti-MyoD, and anti-Myomaker antibodies (Fig. 6e–h). In floxed-control mice, a large number of TRPV2-positive cells were present within myofibre gaps (Fig. 6e, upper panels). These cells were Pax7-positive (Fig. 6f, upper panels). Additionally, MyoD- and Myomaker-positive cells were observed in floxed-control mice (Fig. 6g, h, upper panels). In contrast, the number of TRPV2-positive cells in myofibre gaps was significantly lower among *TRPV2*-*Pax7*-cKO mice (Fig. 6e, lower panels). The number of Pax7-, MyoD-, and Myomaker-positive MuSCs was also substantially reduced in *TRPV2*-*Pax7*-cKO mice (Fig. 6f–h, lower panels). These findings indicate that TRPV2 facilitates muscle regeneration by promoting MuSC activation, proliferation, and the early stages of myogenesis in response to cardiotoxin-induced injury.

To quantitatively assess the timing of *TRPV2* mRNA expression, we performed real-time polymerase chain reaction (PCR) analysis using cardiotoxin-injected TA muscle isolated from floxed mice (Fig. 6i, original data-4). *TRPV2* mRNA was highly expressed from Day 1, whereas *MyoD*, a marker of myogenic commitment and MuSC activation [6], peaked on Day 3 (Fig. 6i, left and middle panels). *Myomaker*, a muscle-specific membrane protein essential for myogenesis [25], also peaked on Days 3–5 (Fig. 6i, right panels). This finding supports the idea that TRPV2 in MuSCs regulates the early stages of myogenic progression during the muscle regeneration process in response to cardiotoxin.

### Impairment of mechanical load-induced hypertrophy in TRPV2-deficient mice

**Fig. 7:**
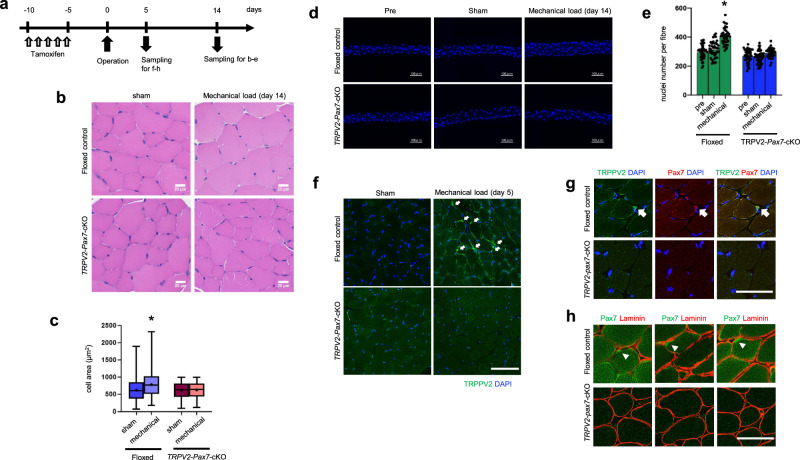
Impaired hypertrophic response in MuSC-specific TRPV2-deficient mice. **a** Timeline for TRPV2-deficiency induction, surgical intervention, and tissue sampling. **b** Representative haematoxylin and eosin staining of plantaris (PLT) muscle. **c** Quantification of cross-sectional area from paraffin-embedded PLT muscle sections (*n* = 1331–1492 cells from three PLT muscles per group). Centre line = median; + = mean; box limits = upper and lower quartiles; whiskers = minimum and maximum. **d** Representative DAPI staining in isolated myofibres from floxed and cKO PLT muscle. **e** Quantification of the number of nuclei per myofibre. **f** Representative immunofluorescence staining of TRPV2 (green) and DAPI (blue) in PLT muscle from floxed and cKO mice. Arrows indicate TRPV2^+^ cells. **g** Representative immunofluorescence staining of TRPV2 (green), Pax7 (red), and DAPI (blue) in PLT muscle from floxed and cKO mice. Arrows indicate double-positive cells. **h** Representative immunofluorescence staining of Pax7 (green), laminin (red), and DAPI (blue) in EDL muscle. Arrowheads indicate Pax7^+^ cells. Data are presented as mean ± s.e.m. *P < 0.05 vs. vehicle-treated floxed MuSCs (Tukey–Kramer test). Scale bars: 20 µm (**b**), 50 µm (**g**, **h**),100 µm (**d**, **f**).

Skeletal muscle undergoes hypertrophic growth in response to mechanical loading [2]. To examine the effect of TRPV2 deficiency on mechanically induced hypertrophy, we subjected the plantaris (PLT) muscle of tamoxifen-treated *TRPV2*-*Pax7*-cKO mice to synergist ablation for 2 weeks (Fig. 7a). Mechanical loading-induced muscle hypertrophy in floxed-control mice but not *TRPV2*-*Pax7*-cKO mice (Fig. 7b, c). To calculate the number of nuclei contained within the muscle fibres, PLT muscles were stained with 4’,6-diamidino-2-phenylindole (DAPI) (Fig. 7d). In floxed-control mice, mechanical loading resulted in greater myofibre thickness and a substantial increase in the number of nuclei per fibre (Fig. 7d, e). However, in *TRPV2*-*Pax7*-cKO mice, neither myofibre thickness nor the number of myonuclei changed in response to mechanical loading (Fig. 7d, e). These findings suggest that TRPV2 in MuSCs is essential for mechanically induced hypertrophy.

In cardiomyocytes and smooth muscle cells, TRPV2 facilitates mechanical stimulus-dependent Ca^2+^ responses [17, 21]. To determine whether TRPV2 expression is upregulated during the early stages of the muscle hypertrophic response, we examined its expression in muscle tissue (Fig. 7a, f). At 5 days postoperatively, floxed-control mice exhibited strong TRPV2 expression in regions of MuSCs within myofibre gaps (Fig. 7f, white arrows). In *TRPV2*-*Pax7*-cKO mice, TRPV2 expression was absent within these regions, even under mechanical loading conditions (Fig. 7f, lower panels). Figure 7g shows that cells with high TRPV2 expression in cells attached to myofibre of mechanically loaded floxed-control mice were also Pax7-positive (Fig. 7g, upper panels). However, in *TRPV2*-*Pax7*-cKO mice, neither TRPV2- nor Pax7-positive cells were detected in myofibre gaps, even after mechanical loading (Fig. 7g, lower panels). Furthermore, in mechanically loaded myofibres of floxed-control mice, Pax7-positive cells were localised within MuSC regions inside the basement membrane (Fig. 7h). These findings suggest that TRPV2 promotes Pax7 expression in MuSCs in response to mechanical loading.

### Mechanical stress-dependent Ca^2+^ response in MuSCs is TRPV2-dependent

**Fig. 8:**
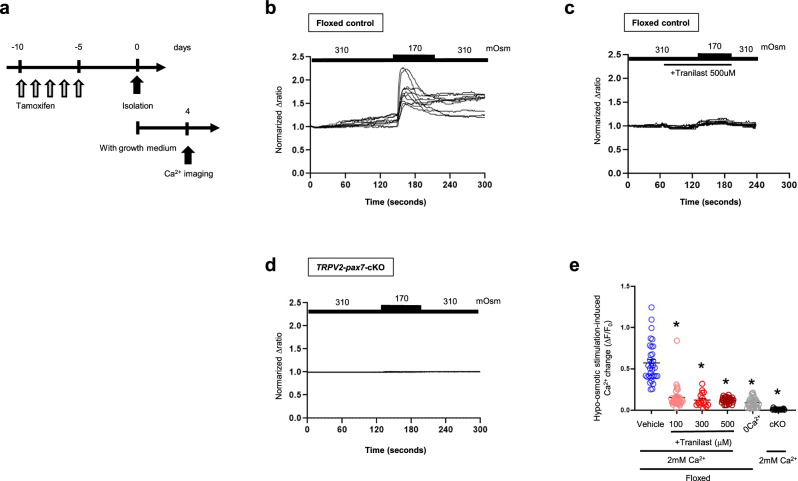
Increase in Pax7-positive MuSCs after AAV-TRPV2 treatment. **a** Timeline for TRPV2-deficiency induction, cell isolation, and Ca^2+^ imaging. **b**, **c** Hypo-osmotic stimulation-induced Ca^2+^ response in floxed MuSCs with or without 500 μM tranilast. **d** Hypo-osmotic stimulation-induced Ca^2+^ response in TRPV2-deficient MuSCs. **e** Effects of TRPV2 inhibition or TRPV2 elimination (*n* = 17–35 cells from three mice). Data are presented as mean ± s.e.m. *P < 0.05 vs. vehicle-treated floxed MuSCs (Tukey–Kramer test).

To determine whether MuSCs exhibit a Ca^2+^ response to mechanical stress and whether this response is TRPV2-dependent, we performed Ca^2+^ imaging experiments on MuSCs after 4 days of culture in growth medium (Fig. 8a). Because proliferating MuSCs are spherical and cannot be subjected to direct stretching stimuli, we analysed Ca^2+^ responses under membrane deformation induced by hypo-osmotic stimuli. MuSCs derived from EDLs of floxed-control mice showed a Ca^2+^ response to hypo-osmotic stimulation, which was abolished after administration of tranilast, a TRPV2 inhibitor (Fig. 8b, c). In contrast, MuSCs from *TRPV2*-*Pax7*-cKO mice showed no response to hypo-osmotic stimulation (Fig. 7d, e). These findings indicate that TRPV2 is essential for mechanical-stimulation-induced Ca^2+^ responses in MuSCs.

## Discussion

The morphological and functional remodelling of skeletal muscle in response to physical activity, ageing, and injury repair is supported by mature multinucleated myofibres and MuSCs residing around the myofibres [1, 2, 5, 6]. Although extensive research elucidated many molecular mechanisms governing the proliferation and activation of mitotically quiescent MuSCs during muscle remodelling [1, 2, 5, 6], the trigger mechanisms that induce MuSC activation remain unclear. This study revealed that TRPV2 in MuSCs is crucial for muscle remodelling, including muscle regeneration and mechanical loading-induced hypertrophy. In cultured MuSCs proliferating at the periphery of isolated myofibres, TRPV2 was expressed in Pax7-positive cells, and these cells displayed a TRPV2-dependent Ca^2+^ response. TRPV2-deficient MuSCs exhibited a substantial reduction in proliferative capacity, a lower number of Pax7-positive cells, and impaired myogenic fusion. MuSC-specific TRPV2-deficient mice demonstrated significantly impaired regeneration after cardiotoxin-induced muscle injury and reduced hypertrophic responses to mechanical loading. Furthermore, MuSCs showed a TRPV2-dependent Ca^2+^ response to mechanical stress. These findings indicate that TRPV2 in MuSCs is essential for promoting the early stage of MuSC function during muscle remodelling. TRPV2 is expressed in both mature myofibres and mitotic MuSCs (Figs. 1 and 3). In the present study, TRPV2 deletion in adult mouse MuSCs did not affect muscle tissue morphology (Fig. 2i) or mouse behaviour, suggesting that MuSC-expressed TRPV2 has a minimal role in muscle function under physiological conditions. In addition, the number of MuSCs sorted by FACS is at the same level in tamoxifen-treated flox and TRPV2-*Pax7*-cKO mice (Fig. 2f), indicating that TRPV2 does not contribute to the maintenance of MuSC numbers. These results are consistent with the observation that the number of Pax7-positive MuSCs in adult muscle (10 weeks old) treated with tamoxifen at the juvenile stage (5 weeks old) did not differ between the control and *TRPV2-Pax7*-cKO groups (Fig. 2g, h). Furthermore, TRPV2 expression was barely detectable in MuSCs in both groups (Supplementary Fig. S1). However, TRPV2 was strongly expressed in a subset of skeletal muscle cells undergoing fusion in postnatal Day 1 mice (Supplementary Fig. S2). Here, our study showed that TRPV2 in MuSCs is crucial for the early stage of myogenic progression. Tranilast-mediated inhibition of TRPV2 activity also suppressed muscle fusion (Supplementary Fig. S3). The elimination of TRPV2 during embryonic and early postnatal myogenesis may substantially impact muscle physiology by disrupting myogenesis and skeletal muscle differentiation. Systemic knockout mice lacking the functional domain of TRPV2 exhibit perinatal lethality, such that ~2.5% of offspring survive on a C57BL/6 background [26]. This outcome may partly reflect defects in myogenic progression. On a BL6129SF2/J background, the survival rate increases to ~15%, and no significant abnormalities in reproduction or external appearance are observed, other than a slight reduction in body weight [26]. These observations suggest the presence of compensatory mechanisms that mitigate the phenotypic effects of TRPV2 deficiency. Additional studies of TRPV2-deficient mice that strictly control targeted cells for depletion and timing are required to elucidate the roles of TRPV2 in skeletal muscle cell development, differentiation, muscle physiology, and pathology.

TRPV2 deletion in MuSCs significantly impaired both cardiotoxin-induced muscle regeneration and mechanical loading-induced hypertrophy (Figs. 6 and 7). The extent of myofibre damage differs between these models, and the niche environments for MuSCs are distinct [6]. Therefore, the mechanisms triggering activation of mitotically quiescent MuSCs likely differ between these models. In regenerating muscle, the loss of components of the MuSC niche, including myofibres, may partially contribute to quiescent MuSC activation [27, 28]. In contrast, the MuSC niche is preserved in overloaded muscle [29]. The activation and proliferation of MuSCs in this context would require either an increase in factors promoting MuSC function through myofibre-dependent mechanotransduction or the direct sensing of mechanical forces by MuSCs themselves. A novel aspect of this study is the finding that MuSCs directly sense mechanical stress, in a TRPV2-dependent manner (Fig. 8). Our experiments showed that skeletal muscle from MuSC-specific *TRPV2* cKO mice exhibited a substantially reduced number of pax7-positive cells and proliferation of MuSCs in both cardiotoxin-induced muscle regeneration and mechanical loading-induced hypertrophy models (Figs. 6 and 7). A similar decrease in proliferation was observed in isolated myofibre cultures lacking direct contact with the basement membrane, where pax7-positive cells were greatly reduced (Figs. 3 and 4). These findings suggest that TRPV2 plays a central role in the early stage of MuSC function for myogenesis and proliferation, independent of myofibre integrity or niche status. In future studies, the role of TRPV2 in MuSC proliferation and fusion during muscle remodelling could be examined in greater detail by isolating and culturing MuSCs from damaged and hypertrophic muscle fibres via FACS. In this study, DAPI-positive cells lacking Pax7 expression were observed on Day 3 in the control EDL culture (Fig. 3a). To characterise these cells, double staining with anti-Pax7 and anti-MyoD antibodies revealed the presence of Pax7^−^/MyoD^+^ cells on Day 3, although they were absent on Day 2 (Supplementary Fig. S4). Thus, the number of MuSCs on fibres is considerably reduced after TRPV2 deletion. A small number of Pax7^−^/MyoD^−^ cells were also detected; we aim to elucidate their identity in future studies.

Hepatocyte growth factor, basic fibroblast growth factor, and insulin-like growth factor-1 (IGF-1) are potential regulators of MuSC proliferation during muscle regeneration [30–33]. IGF-1 promotes the translocation of TRPV2 to the plasma membrane [18, 34] and enhances its activity [34]. Because the MuSC-specific TRPV2-deficient mice used in this study retain intact myofibres, the levels of IGF-1 derived from damaged myofibres and the surrounding niche environment should be comparable to those in controls. TRPV2 elimination reportedly downregulates the IGF-1 receptor/phosphoinositide 3-kinase pathway in cardiomyocytes [21]. In this context, the impaired muscle regeneration we observed in TRPV2-deficient MuSCs may result from downregulation of IGF-1 receptor signalling in MuSCs. It is also possible that MuSCs regulate IGF-1 production in an autocrine manner, depending on muscle conditions. For example, in cardiomyocytes and ventricular fibroblasts, IGF-1 is secreted extracellularly in response to mechanical stimuli [35, 36]. We previously showed that the IGF-1 secretion of cardiomyocytes depends on TRPV2 [21]. Even during muscle regeneration not directly triggered by mechanical stress, the physical environment surrounding MuSCs is likely to be dynamically altered by myofibre degradation [37]. Although the specific source of IGF-1 during muscle regeneration remains unclear, TRPV2 elimination in MuSCs may lead to the impairment of the regeneration process mediated by IGF-1.

Serum response factor-dependent interleukin-6 paracrine signalling within myofibres activates MuSCs, leading to muscle hypertrophy [38]. Silent mating type information regulation 2 homologue 1 (Sirt1)-dependent humoral factors in myofibres may function as MuSC growth factors [39]. However, the mechanosensor molecules that activate serum response factor or Sirt1 in myofibres remain unknown. In the MuSC-specific *TRPV2* cKO model used in this study, the production and secretion of interleukin-6 or Sirt1-dependent factors are expected to be similar to levels in control mice because myofibres remain intact. Thus, the absence of a mechanical load-dependent hypertrophic response in TRPV2-deficient MuSCs may result from a failure of MuSCs to respond to myofibre stimulation. Alternatively, mechanotransduction in MuSCs may play a pivotal role in the mechanical load-dependent hypertrophic response via TRPV2. These findings suggest that TRPV2 has a crucial function in regulating the myogenic function of MuSC in response to mechanical loading.

In this study, we showed that TRPV2 is involved in the Ca^2+^ response of MuSCs to mechanical stimulation. However, we cannot conclude that TRPV2 directly induces Pax7 expression because no data currently support its role as a primary transducer of mechanotransduction in vivo. Nonetheless, TRPV2 was expressed in Pax7-positive MuSCs under continuous mechanical loading (Fig. 7f–h). When mechanical loading induces TRPV2 expression in MuSCs, the PI3K/Akt pathway may be activated downstream of TRPV2-mediated Ca^2+^ signalling (Figs. 4k and 5e), promoting cell proliferation and contributing to muscle hypertrophy through enhanced MuSC fusion. Thus, TRPV2 may act as an amplifier of the muscle hypertrophy pathway triggered by mechanical loading.

Matrix elasticity reportedly directs stem cell lineage specification, as described by Engler et al. [40]. There is evidence that both biochemical and physical properties of the microenvironment play key roles in cell fate determination [41–43]. However, the molecular identity of mechanosensors in MuSCs remains unclear. Piezo1 channels exert stage-dependent functions in myogenesis and promote MuSC function during muscle regeneration [14]. Additionally, experiments with C2C12 cells showed that PIEZO1-mediated Ca^2+^ influx activates RhoA/ROCK-mediated actomyosin assembly at the lateral cortex of myotubes [44]. Among the TRPV family members, TRPV2 and TRPV4 reportedly respond to hypotonic cell swelling, shear stress, and membrane stretching [15]. TRPV2 is expressed in human cancer stem cells [45–47] and progenitor cells [48]; its overexpression is reported to suppress stemness [49]. Conversely, TRPV2 elimination substantially upregulates cancer stem cell markers while enhancing spheroid and hepatoma colony formation in human hepatoma HEpG2 cells [47]. These experimental results are consistent with our findings that TRPV2 promotes MuSC function and is essential for muscle remodelling in vivo. Considering that TRPV2 functions as a polymodal mechanosensor responding to both chemical and physical stimuli, it may interact with the dynamic microenvironment at various stages of MuSC function to integrate multiple biological signals. Further studies are warranted to identify specific stages at which TRPV2 interacts with other regulatory molecules.

Multiple mechanosensitive ion channels may play critical roles in the activation, proliferation, and differentiation of MuSCs [4, 14]. It is possible that these channels provide functional compensation in the absence of TRPV2. However, in our study, MuSCs lacking TRPV2 failed to exhibit a Ca^2+^ response to mechanical stimuli (Fig. 8d, e). This observation suggests that other channels are unlikely to compensate for the loss of TRPV2 in mediating mechanosensitive Ca^2+^ responses in *TRPV2*-*Pax7*-cKO MuSCs. Recently, the TRPM7 channel, a candidate mechanosensitive ion channel, has been reported to mediate Mg^2+^ influx that promotes MuSC activation [50]. Efforts to clarify the distinct roles of various mechanosensitive ion channels in MuSC activation, proliferation, and differentiation, as well as their potential functional interactions, remain important for future studies.

This study suggests that the number of Pax7-positive cells in muscle remodelling may be regulated by TRPV2. An increased number of Pax7-positive cells has also been observed in the muscle of patients with Duchenne muscular dystrophy and dystrophin-deficient *mdx* mice [51, 52]. However, functional defects in dystrophic satellite cells impair asymmetric cell division and myogenic commitment, leading to reduced muscle regeneration in Duchenne muscular dystrophy patients [53]. Because TRPV2 expression may be upregulated in MuSCs of *mdx* mice as a compensatory mechanism for functional deficits, further studies are necessary to elucidate the mechanisms that underlie TRPV2 upregulation in dystrophic muscle.

This study demonstrated that TRPV2 is essential for MuSC function in muscle remodelling and offered a therapeutic strategy targeting TRPV2 in MuSCs. Although the physiological role of TRPV2 in myogenesis and mature myofibres remains unclear, further functional analyses will clarify its potential as a therapeutic target in muscle pathology.

## Materials and methods

### Animals

The generation of *TRPV2*^*flox/flox*^*;Pax7* ^*CreERT2/*^ (floxed-*TRPV2*) mice was previously described in detail [22]. To produce *TRPV2*^*flox/flox*^*;Pax* ^*CreERT2/+*^ mice, we crossed mice carrying a *TRPV2* ^*flox/flox*^ allele with transgenic mice expressing tamoxifen-inducible MuSC-specific Cre recombinase (*Pax7*^*CreERT2/+*^ mice) [23, 24]. To induce Cre-mediated recombination, 10-week-old *TRPV2-Pax7*-cKO and floxed-*TRPV2* male mice received intraperitoneal injections of tamoxifen (8 mg/kg; Sigma) once daily for 5 consecutive days. Littermates were used to randomise genetic variation. To examine muscle regeneration, cardiotoxin (50 μL of 10 μM) was injected into the TA muscle, and samples were collected after 7 days.

### Synergist ablation surgery

Male mice (10 weeks old) were anaesthetised with a combination of 0.3 mg/kg medetomidine (Zenoaq), 4.0 mg/kg midazolam (Sandoz), and 5.0 mg/kg butorphanol (Meiji Seika Pharma). Mechanical overload of the plantaris muscle was induced by bilateral surgical ablation of the tendons of the gastrocnemius and soleus muscles. After a midline incision had been made in the skin of the hindlimb, the distal tendons of the gastrocnemius and soleus muscles were severed. The incision was then closed using a 7-0 silk suture (Matsuda Ika Kogyo). In the sham-operated group, identical skin incisions were made, but the tendons remained intact.

### MuSC isolation via fluorescence-activated cell sorting

Mononuclear cells from uninjured limb muscles were isolated using 0.2% collagenase type II (Worthington Biochemical Corporation), as previously described [54]. These cells were then stained with fluorescein isothiocyanate-conjugated anti-CD31 (BD Pharmingen, 558738, 1:400), anti-CD45 (Ptprc; eBioscience, 11-0451-82, 1:800), and phycoerythrin-conjugated anti-Sca-1 (Ly6a) (BD Pharmingen, 553336, 1:400) antibodies, along with a biotinylated SM/C-2.6 antibody [55], in accordance with an established protocol [56]. Subsequently, cells were incubated with streptavidin-labelled allophycocyanin (BD Biosciences) on ice for 30 min and re-suspended in phosphate-buffered saline containing 2% foetal calf serum and 2 µg/ml propidium iodide. Cell sorting was performed using a FACS Aria II flow cytometer (BD Immunocytometry Systems). Debris and dead cells were excluded based on forward scatter, side scatter, and propidium iodide gating. Data were acquired using FACSDiva software (BD Biosciences).

### Real-time PCR analysis

Total RNA was extracted from TA muscles using TRIzol LS and a QIAGEN RNeasy Micro Kit (QIAGEN), in accordance with the manufacturer’s instructions. cDNA synthesis was performed using a QuantiTect Reverse Transcription Kit (QIAGEN), as previously described [57].

### Single myofibre isolation and culture

Single myofibres were isolated from EDL muscles using an established protocol [30, 58]. The isolated myofibres were incubated in DMEM containing 0.5% collagenase type I (Worthington Biochemical Corporation) at 37 °C for 1 h. Myofibres were then mechanically separated from the muscle by gentle flushing and cultured in GlutaMAX™ (Gibco) supplemented with 30% foetal bovine serum, 1% chicken embryo extract (Life Sciences), 10 ng/mL basic fibroblast growth factor (ORIENTAL YEAST CO., Ltd.), and 1% penicillin–streptomycin (Fujifilm Wako Pure Chemical Corporation). To induce myogenic fusion, the culture medium was replaced on Day 5 with GlutaMAX™ containing 5% horse serum and 1% chicken embryo extract; cells were incubated for an additional 3 days at 37 °C. Adeno virus (Ad) vector encoding mouse TRPV2 under the CMV promoter was constructed by Vector Builder (Yokohama, Kanagawa, JAPAN). Ad vector encoding Cre-recombinase under the CMV promoter was purchased from SignaGen Laboratories (Frederick, MD, USA). Ad-TRPV2 (6.79 × 10^8^ IFU/ml), Ad-Cre (1 × 10^6^ IFU/ml), or 100 μM tranilast was added at the time of medium replacement. To obtain sufficient cell numbers for protein expression analysis, cells cultured for 6 days were subjected to immunoblotting. Because Ca^2+^ imaging experiments involve fluid exchange, proper cell adhesion to the culture dish was required; thus, cells cultured for 4 days were used for Ca^2+^ imaging.

### Measurement of intracellular Ca^2+^ in MuSCs

Changes in intracellular Ca^2+^ were assessed in cultured MuSCs loaded with 2 µM fura-2 acetoxymethyl ester (fura-2) for 30 min at 37 °C. Cells were maintained in standard Tyrode’s solution under continuous flow using a microperfusion system, as previously described [59]. Fura-2-loaded cells were alternately excited at 340 and 380 nm using a Lambda DG-4 Ultra High-Speed Wavelength Switcher (Sutter Instruments) coupled to an inverted IX71 microscope equipped with a UApo ×20/0.75 objective lens (Olympus). Fura-2 fluorescence signals were recorded using an ORCA-Flash 2.8 camera (Hamamatsu Photonics) and analysed by ratiometric fluorescence imaging using MetaFluor software (version 7.7.5.0; Molecular Devices).

### Antibodies

The following antibodies were used for immunostaining and immunoblotting analyses: anti-TRPV2 (Sigma; HPA044993, 1:1000 dilution), anti-Pax7 (Abcam; ab34360, 1:1000 dilution), anti-glyceraldehyde-3-phosphate dehydrogenase (GAPDH; Abcam; EPR16891, 1:1000 dilution), anti-laminin (Abcam; ab11576, 1:1000 dilution), Akt (Cell Signaling Technology; 9272, 1:1000 dilution), P-Akt (Cell Signaling Technology; 4060, 1:1000 dilution), PI3K (Cell Signaling Technology; 4249, 1:1000 dilution), myoD (DSHB; D7F2-c, 1:1000 dilution), and anti-Ki67 (Proteintech; 28074-1-AP, 1:600 dilution). Immunoreactive bands were detected using an enhanced chemiluminescence detection system (Amersham Biosciences Corp.) and a Luminescent Image Analyzer (LAS3000; Fujifilm).

### Histological analysis

Skeletal muscles were excised, immediately fixed with buffered 4% paraformaldehyde, embedded in paraffin, and sectioned at 4 µm. Samples were stained with haematoxylin and eosin or processed for immunocytochemistry.

### Immunocytochemistry

Five-micrometre frozen muscle sections embedded in OCT compound (Tissue-Tek) were permeabilised using 0.1% Triton X-100 and incubated with primary antibodies. Alternatively, cultured MuSCs immobilised on collagen-coated glass slides were fixed with 4% paraformaldehyde for 15 min at room temperature, permeabilised using 0.1% Triton X-100, and stained with primary antibodies. Samples were then incubated with Alexa Fluor 488-conjugated anti-rabbit IgG (A11008, Life Technologies) or Alexa Fluor 488-conjugated anti-mouse IgG (A11001, Life Technologies). Imaging was performed using a confocal microscope (Fluoview FV1000, Olympus) mounted on an Olympus IX81 epifluorescence microscope with a UPlanSApo ×60/1.35 oil immersion objective lens (Olympus).

### Immunoblotting

Cultured MuSCs were homogenised using a Hiscotron homogeniser (NITI-ON) in RIPA buffer, as previously described [60]. Lysates were centrifuged at 12,000×*g* for 20 min. The supernatant was analysed by immunoblotting, in accordance with an established protocol [21]. Immunoreactive bands were visualised using a chemiluminescence detection system (PerkinElmer) and a LAS3000 Luminescent Image Analyzer (Fujifilm).

### Data analysis

Data were analysed by individuals blinded to genotype, drug treatment, and surgical procedure. All data presented were reproducible in at least three independent experiments. Results are expressed as the mean ± standard error of the mean (s.e.m.). Paired data were evaluated via Student’s *t*-test. For multiple comparisons, we performed analysis of variance followed by Tukey–Kramer tests where appropriate. Calculations were carried out using GraphPad Prism version 9. Image analysis and quantification were performed using ImageJ. The numerical source data are provided in the Original Data file. *P*-values < 0.05 were considered statistically significant.

## Supplementary information

Supplemental data

## Data Availability

The datasets generated during and/or analysed during the current study are available from the corresponding author on reasonable request.
