# Supplementary material for: TRPV2 in muscle satellite cells is crucial for skeletal muscle remodelling
Source: Cell Death Dis. 2025 Dec 15;16(1):888. doi: 10.1038/s41419-025-08242-3 (PMC12706003; doi:10.1038/s41419-025-08242-3)
Supplement: Supplementary file 1 — Supplemental data [file 41419_2025_8242_MOESM1_ESM.pdf]

Supplementary Figures

TRPV2 in muscle satellite cells is crucial for skeletal muscle remodelling

Yanzhu Chen[1,2], Kimiaki Katanosaka[3], Makoto Shibuya[1], Yubing Dong[1,2], Lidan Zhang[4,5], Motoi Kanagawa[6], So-ichiro Fukada[4], Keiji Naruse[1], Yuki Katanosaka[1,2]

[1]Department of Cardiovascular Physiology, Graduate School of Medicine, Dentistry and Pharmaceutical Sciences, Okayama University, Okayama, Japan
[2]Department of Pharmacy, Kinjo Gakuin University, Nagoya, Aichi, Japan
[3]Department of Biomedical Sciences, College of Life and Health Sciences, Chubu University, Kasugai, Aichi, Japan
[4]Laboratory of Stem Cell Regeneration and Adaptation, Graduate School of Pharmaceutical Sciences, Osaka University, 1-6 Yamadaoka, Suita, Osaka 565-0871, Japan
[5]Center for Medical Epigenetics, School of Basic Medical Sciences, Chongqing Medical University, Chongqing, 40016, China.
[6]Department of Cell Biology and Molecular Medicine, Ehime University Graduate School of Medicine, Toon, Ehime 791-0295, Japan

Corresponding author: Yuki Katanosaka, Department of Pharmacy, Kinjo Gakuin University, Omori 2-1723, Moriyama-ku, 2-5-1, Nagoya city, Aichi 4638521, Japan. Tel.: 81-52-798-7483. E-mail: katanosaka@kinjo-u.ac.jp

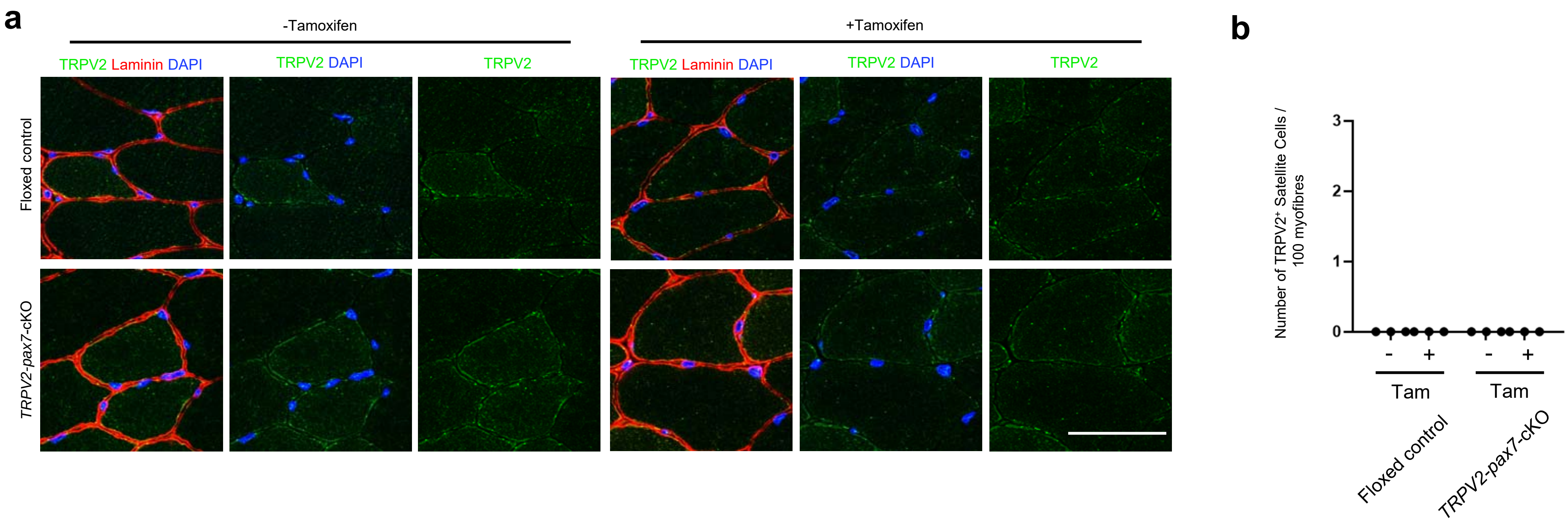

Supplementary Fig. 1: Expression of TRPV2 in MuSCs in muscle tissue under physiological conditions. Representative image of TRPV2 (green), Laminin (red) and DAPI (blue) expression in MuSCs in tibialis anterior (TA) muscle of 10-week-old mice. Scale bar, 50 μm.

Fig. S1 Chen at. el.

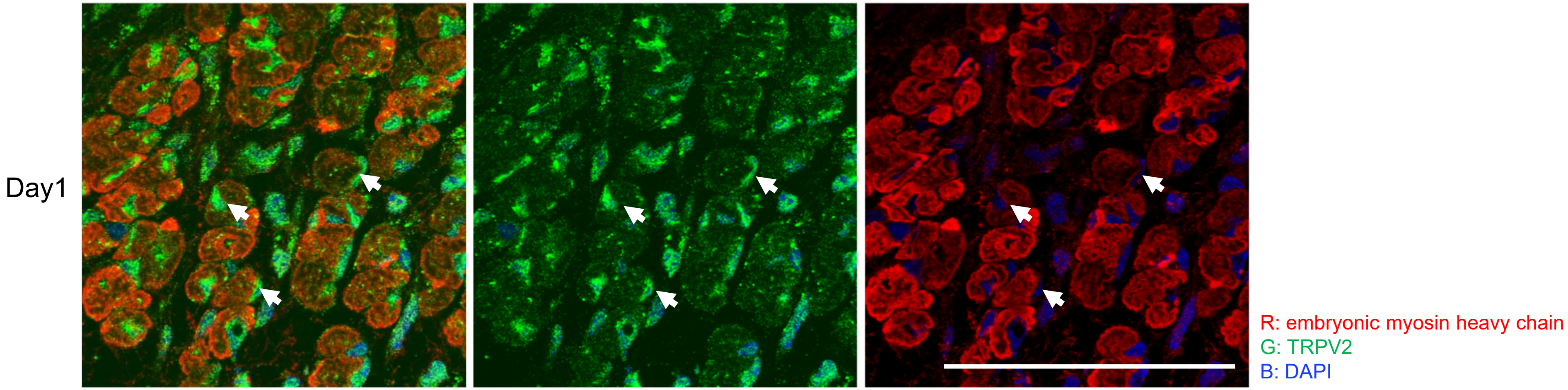

Supplementary Fig. 2: Expression of TRPV2 in small growing cells associated with postnatal day 1 muscle fibres
Representative triple staining of anti-TRPV2 (green), anti-embryonic myosin heavy chain antibody (red) and DAPI (blue) in muscle of Floxed-mice. Scale bar, 100 μm.

Fig. S2 Chen at. el.

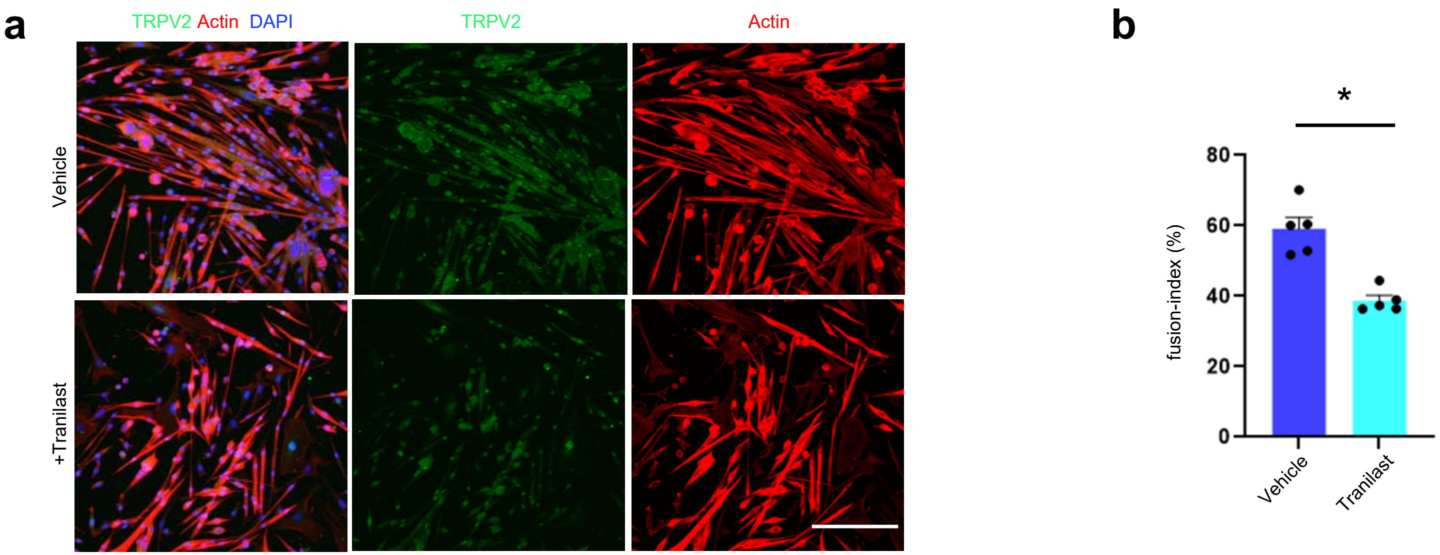

Supplementary Fig. 3: Effects of tranilast in the fusion of MuSCs.
(a) Representative triple staining of anti-TRPV2 (green), phalloidin (red) and DAPI (blue) in MuSCs with 200 μM tranilast. Scale bar, 200 μm.
(b) fusion-index. Data are mean ± s.e.m. *P<0.05 between indicated groups based on a Student's *t*-test.

Fig. S3 Chen at. el.

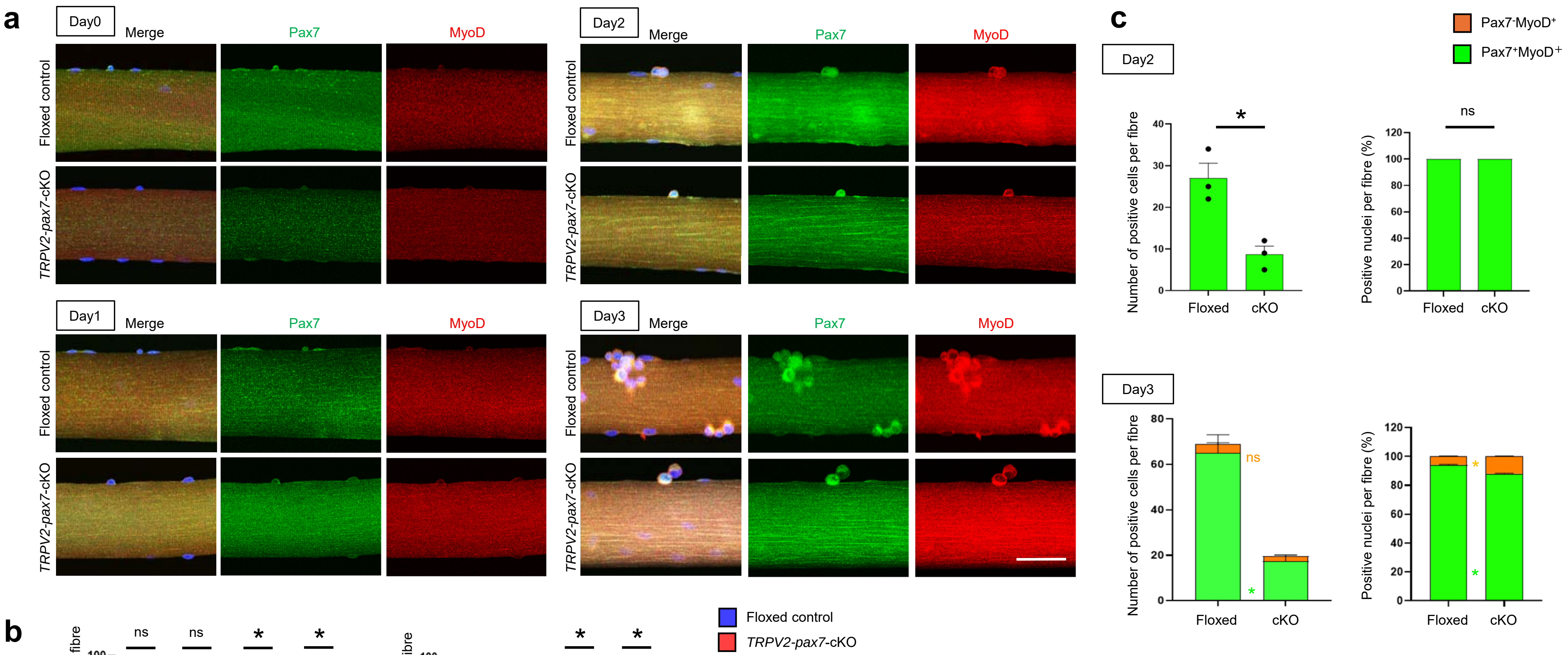

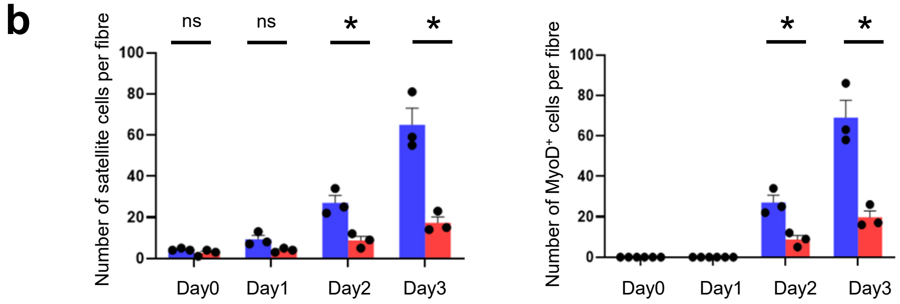

Supplementary Fig. 4: Decreased in MyoD-positive MuSC by TRPV2-deficiency.
(a) Representative immunofluorescence staining of Pax7 (green), MyoD (red), and DAPI (blue) in MuSCs derived from myofibres isolated from the extensor digitorum longus (EDL) muscle of tamoxifen-treated floxed and cKO mice after 3 days in culture. **(b)** Quantification of satellite cell numbers (Pax7$^+$ cells) and MyoD$^+$ cells in cultured floxed and TRPV2-deficient MuSCs. (c) Number of positive cells and percentage of character. Data are presented as mean ± s.e.m. *P<0.05 between the indicated groups (Student's *t*-test). Scale bar, 50 µm.

Fig. S4 Chen at. el.
